# Modified Roux-en-Y gastric bypass surgery avoids complications in mice

**DOI:** 10.1371/journal.pone.0323706

**Published:** 2025-05-15

**Authors:** Dan Tong, Jie Xiang, Peng Gao, Zhiming Zhu, Zongshi Lu

**Affiliations:** 1 Department of Hypertension and Endocrinology, Center for Hypertension and Metabolic Diseases, Daping Hospital, Army Medical University, Chongqing Institute of Hypertension, Chongqing, China; 2 Chongqing Institute for Brain and Intelligence, Chongqing, China; National Healthcare Group, SINGAPORE

## Abstract

**Background:**

Roux-en-Y gastric bypass（RYGB）surgery delivers an improvement in obesity and obesity-related risks. However, due to the limited operational space in the abdominal cavity of mice, the technical complexity of RYGB surgery and the postoperative complications hinder its mechanism research. The aim was to develop a device that makes it easier to anastomose the esophagus to the jejunum.

**Methods:**

We have invented a simple gastrointestinal anastomosis auxiliary device consisting of a rigid front end and a flexible rear end. Thirty male C57BL6J mice were subjected to RYGB with an auxiliary device. Postoperative recovery and survival status of mice were evaluated using body weight, food intake, body fat, and glucose tolerance.

**Results:**

Based on the RYGB surgical methodology reported in previous literature, the anastomosis device described in this article assists in end-to-end anastomosis of the esophagus and jejunum, which reduces surgical difficulty and time. CT scan results revealed that, following a short - term recovery period after mRYGB surgery, no leakage or stenosis was detected at the anastomotic site in the mice. Moreover, after postoperative recovery, there was no significant difference in food intake, weight and body fat distribution compared with Sham mice, but the glucose tolerance of mRYGB mice was significantly improved.

**Conclusions:**

Our modified RYGB surgical method can effectively avoid the problems of anastomotic leakage and stenosis in mice and improve long-term quality of life.

## Introduction

Metabolic surgery has emerged as one of the most effective therapeutic interventions for obesity and diabetes. Among them, Roux-en-Y gastric bypass (RYGB) and vertical sleeve gastrectomy (VSG) are the most popular metabolic surgical approaches in clinical practice [1]. Compared with VSG surgery, RYGB demonstrates a superior effect in weight loss and alleviates obesity-related complications, as it can reduce total body weight by 40% and ameliorating more than 80% of type 2 diabetes mellitus (T2DM) symptoms [2–4]. As an invasive therapeutic modality, the dissemination of RYGB surgery is impeded by elements including surgical risks and patients’ cognitive limitations. This underscores the critical need for elucidating the underlying mechanisms of metabolic surgery to facilitate the development of non-invasive therapeutic alternatives [5].

Among all animal models, mice assume an irreplaceable role in the basic research of metabolic diseases, and their disease models and conditional gene manipulation models are widely used [6].However, in the course of our actual operative procedures, the restricted operating space is likely to be one of the primary factors contributing to the low success rate and the high incidence of postoperative complications. Current mouse RYGB models are categorized into three types: RYGB-small pouch [7,8], RYGB-fundus [9]and RYGB-esophagus [8,9]. Based on Stevenson’s findings, the mortality rates of the RYGB-small pouch, RYGB-fundus, and RYGB-esophagus models are 17%, 50%, and 20% respectively [10]. Among these, the RYGB-small pouch model developed by Hao et al. represents a significant advancement, demonstrating an exceptionally low surgical mortality rate approaching zero [7,11]. Moreover, Hao’s method was successfully replicated in an independent laboratory by Stevenson et al [10,12,13]. While this model closely replicates the classical human RYGB procedure, the level of its technical complexity remains considerable. He et al. reported that even experienced surgeons need approximately four years of practice to reduce mortality rates from 81% to 22% [14], highlighting the urgent need for simplified and more efficient RYGB modeling techniques in basic research.

In order to tackle these technical challenges, our research group has repeatedly explored and has invented a simple and effective surgical auxiliary device, which consists of a rigid front end and a flexible rear end. In the RYGB-esophagus model, after ligation of the stomach above the cardia, the rigid part is implanted into the esophagus, while the flexible part is inserted into the jejunum. This surgical procedure can be performed in a confined space, effectively narrowing the diameter discrepancy between the esophagus and jejunum. This design not only facilitates visual suturing, which helps prevent anastomotic leakage, but also integrates a tubular lining to alleviate postoperative stenosis. The protocols and anastomotic devices provided here are intended to offer reliable, complication-free, effective techniques for the mouse models of RYGB that can be easily followed by scientists of different backgrounds.

## Materials and methods

### Ethics statement

All experimental procedures were approved by the institutional animal care and research advisory committee at Daping Hospital, Army Medical University (AMUWEC20237044).

### Animals

The 10-week-old male C57BL/J mice weighing approximately 26 grams were obtained from the Charles River. All mice were housed in cages at a controlled temperature (22 ± 1°C) and relative humidity (55 ± 5%) in a 12-h light/12-h dark cycle. They were supplied with standard laboratory chow and tap water ad libitum. On postoperative week 8 or at the end of the experiment, the mice were euthanized by cervical dislocation.

### Auxiliary assistance device

The auxiliary assistance device is a tube consisting of a rigid and flexible part. The rigid part is a stainless steel tube with a diameter of 0.4mm and a length of 1.5 cm. The flexible part is a silicone tube with an inner diameter of 0.3mm and an outer diameter of 0.8mm. The length of the flexible part is 2 cm. Due to the difference in diameter between the two parts, the stainless steel tube can be tightly connected to the silicone tube to form the device.

### Surgical procedures

The surgical instruments were sterilized at high temperatures before use. The surgery was performed on the mice when they were ten weeks old and weighed approximately 26 grams. The mice were fasted overnight before the surgery but had free access to water. After a 14-hour fast, mice were injected with 5mg penicillin before surgery. Then mice were anesthetized with isoflurane and a warming pad during the surgery (VME animal anesthesia machine, Matrx Company, USA). After shaving the abdomen of the mouse, the skin is cleaned with povidone-iodine. Cover the entire mouse with a sterile drape, exposing only the surgical area.

### RYGB surgery

After anesthesia, an appropriate opening was made in the middle and upper abdomen to fully expose the surgical field. The esophagus and stomach were separated from the surrounding ligaments and blood vessels, and the stomach was clipped at the cardia. The stomach was transected at the upper part of the clamp and the gastric stump was interrupted sutured with a 9–0 silk. The jejunum was transected at 4–6 cm from the Treitz’s ligament, and the distal jejunum was end-to-end anastomosed with the lower esophagus by a 9–0 silk interrupted suture. A small incision was cut 4–6 cm away from the gastrointestinal anastomosis. Then the jejuno-jejunostomy was performed with a 9–0 silk interrupted suture between the aforementioned incision and the proximal jejunum. The intestine was moistened with warm saline during the procedure. The abdominal cavity was closed with a 6–0 silk interrupted suture.

### Modified RYGB (mRYGB) Surgery

**Fig 1:**
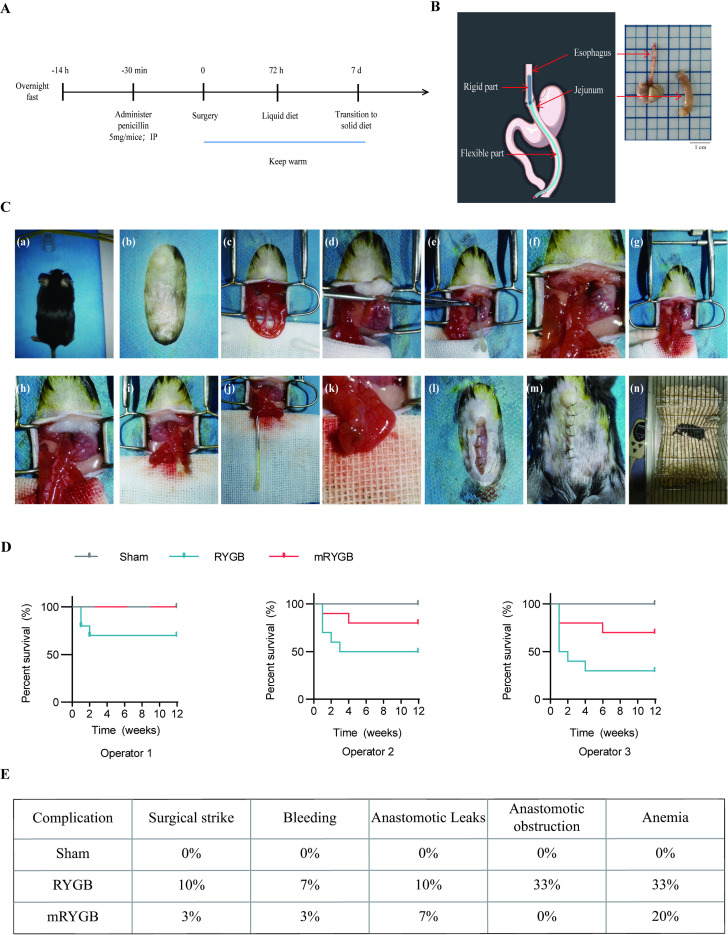
RYGB in the mice. A, Experimental flow chart. Mice were fasted overnight before surgery and injected with penicillin 30 min before surgery. After surgery, mice were placed on a heating pad, followed by a fluid diet 72h later and a solid diet 7 days later. B, Schematic representations of anastomotic device. The right panel showed the esophagus and jejunum. C, mRYGB (RYGB with anastomotic devices) in the mice. (a,b) After scraping and disinfecting the abdominal skin, the mice were draped to expose the surgical. (c) A retractor was placed to expose the abdomen. At the jejunum 4–6 cm away from the ligament of Treitz, an indicative suture was placed to mark the end of the Roux limb, and then the jejunum was transected at this marked point. (d) The perigastric ligaments and blood vessels were separated and ligated to release the stomach. (e-h) The gastroesophageal junction was transected at the cardia. The rigid part of the XL anastomotic device was inserted into the esophagus, and the flexible part into the intestinal cavity. Along the surface of the anastomotic device, the lower esophagus and the distal jejunum were end-to-end anastomosed. (i,j) The XL anastomotic device was taken out at a small incision 4–6 cm away from the gastrointestinal anastomosis. (k) Then the jejuno-jejunostomy was performed between the incision mentioned above and the proximal jejunum. (l,m) The muscle layer and skin were sutured separately to close the abdominal cavity. (n)After surgery, mice were injected with meloxicam (1 mg/kg) and then placed on a heating pad. D, Percent survival of sham, RYGB and mRYGB surgery performed by three surgeons. Mice were weight-matched and performed for Sham, RYGB, and mRYGB surgery (n = 10). E, Complication of sham, RYGB, and mRYGB surgery (n = 30).

The operation method of mRYGB is similar to that of RYGB, but the anastomotic device is used to assist the esophagojejunostomy. The rigid part was inserted into the esophagus, and the flexible part into the intestinal cavity. Along the surface of the anastomotic device, the lower esophagus and the distal jejunum were end-to-end anastomosed. The anastomotic device was taken out at a small incision 4–6 cm away from the gastrointestinal anastomosis. Other surgical procedures are the same as the described in RYGB. The detailed surgical steps can be seen in Fig 1C.

### Sham surgery

For the sham operation, the esophagus and stomach were separated as described in RYGB and mRYGB. After cutting off the above anastomotic site, the in situ anastomosis was performed.

### Postoperative care

Mice were placed on a heating pad (37°C) immediately after surgery. For the first 24 h after surgery, mice were subcutaneously injected with meloxicam (1 mg/kg) every 8 h and fully fast (no food or water). For the first 72h, the health status of mice must be observed twice a day. Mice were free to drink water 24h after surgery. Mice were fed with a liquid diet (Vital HN Vanilla; Abbott Nutrition, cat. no. 00766) on postoperative day 3 for 4 d. On day 7, transits to solid diet feeding, and during the period before fully transitioning to solid diet, mice were fed a mixture of liquid and solid diet. The detail is as described by a previous report [3].

### Postoperative complication analysis

(1)Surgical strike： Surgery-related mortality；(2)Bleeding： Postoperative bleeding typically occurs when blood vessels behind the esophagus and stomach rupture without complete hemostasis. We diagnosed bleeding in the mice through autopsy；(3)Anastomotic Leaks： Leakage of gastrointestinal contents due to an incomplete anastomosis. We diagnosed anastomotic leaks in the mice through autopsy;(4)Anastomotic Obstruction： Food cannot pass smoothly into the digestive tract due to anastomotic stricture. We diagnosed anastomotic obstruction in the mice through autopsy;(5)Anemia: Excluding other complications, symptoms such as postoperative anorexia, pale extremities, and weight loss in mice are considered indicative of anemia. We diagnosed anemia in mice based on their postoperative behavior.

### Micro-CT analysis

On postoperative week 3, the mice were anesthetized under isoflurane with an air pump throughout the micro-CT process. Before anesthesia, mice were gavaged with 5% BaSO4 solution (BaSO4: CT contrast agent). During the acute imaging procedure, anesthesia was maintained by mask inhalation of isoflurane. At the same time, the researchers observed the anesthesia status of the mice through a built-in camera and adjusted the flow rate of isoflurane on time. The CT was acquired over 360 projections using 70 kV, and 600 μA on a CMOS detector and reconstructed using a modified algorithm. For fat quantification, the visceral and subcutaneous fat in each CT image -were circled based on the difference in CT values, and the visceral and subcutaneous fat were rendered in different colors. Fat volume is evaluated by reconstructing three-dimensional surfaces through rendering [15]. All data were analyzed by the Avatar3 software (PINGSENG Healthcare).

### OGTT and IPGTT

For glucose tolerance tests, animals were fasted overnight (18:00–6:00) and then orally gavaged or intraperitoneally injected with D-glucose at 1.5 g/kg [16]. Blood glucose was measured at the indicated time points using the One-Touch Ultra blood glucose meter (LifeScan) through tail-vein bleeding. IPGTT and OGTT were performed using different batches of animals.

### Hematoxylin-eosin (HE) staining

The esophagojejunal anastomosis site was fixed in 4% paraformaldehyde overnight at 4 °C, then embedded in paraffin before sectioning and cut into 4-μm thick sections. The paraffin sections were stained with hematoxylin-eosin (HE) and the images were taken using a Nikon E200 light microscope.

### Statistical analysis

The results are the mean ± SEM. All analyses were performed with GraphPad Prism9. Unless indicated, comparisons between groups were made using a two-tailed unpaired Student’s t test. Differences were considered significant when *P* values of 0.05 or less. All images shown without biological replicates are representative of at least three independent experiments.

## Result

### mRYGB surgery is more effective and reliable

The surgical procedure is shown in Fig 1A. In the process of constructing the RYGB surgical model, we improved the suturing efficiency by using anastomotic assistance devices, thereby reducing the surgery time by more than half an hour. As shown in Fig 1B, the diameter difference between the esophagus and jejunum of mice was significant, which posed a great challenge for direct surgical suturing. This was also a key factor in the occurrence of anastomotic leakage and stenosis after RYGB surgery in mice. Therefore, we adopted an auxiliary device consisting of a rigid and flexible part (Fig 1B). The rigid part was inserted into the esophagus to expand the esophagus appropriately, while the flexible part was inserted into the intestinal cavity to avoid damage the jejunal epithelium. Using a simple device could bring direct benefits. The size deviation of the dilated esophagus and jejunum would be reduced, which was conducive to more uniform spacing during suturing. In addition, the entire suturing operation was carried out against the surface of the anastomotic device, and the rigid part effectively reduced repeated pulling of the esophagus, thus completely avoiding the narrowing of the anastomotic opening caused by suturing. After the esophagojejunal anastomosis was completed, a suitable small incision was cut on the sidewall of the jejunum 4–6 cm below the anastomosis site, and the anastomotic device was taken out. This incision was then anastomosed with the proximal jejunum, allowing digestive juices such as gastric juice, bile, and pancreatic juice to flow back into the intestinal cavity（Fig 1C）. Fig 1D shows the survival of mice subjected to sham, RYGB, and mRYGB surgery by three surgeons with 10 years, 5 years, and 1 year of surgical experience, respectively. At 12 weeks after surgery, the survival rate of the mRYGB surgery group was approximately 30% higher than that of the conventional RYGB surgery group. mRYGB surgery significantly improved the postoperative survival rate of mice. In addition, the postoperative complications of mRYGB group were reduced（Fig 1E）.

### mRYGB surgery avoids anastomotic leakage and stenosis

**Fig 2:**
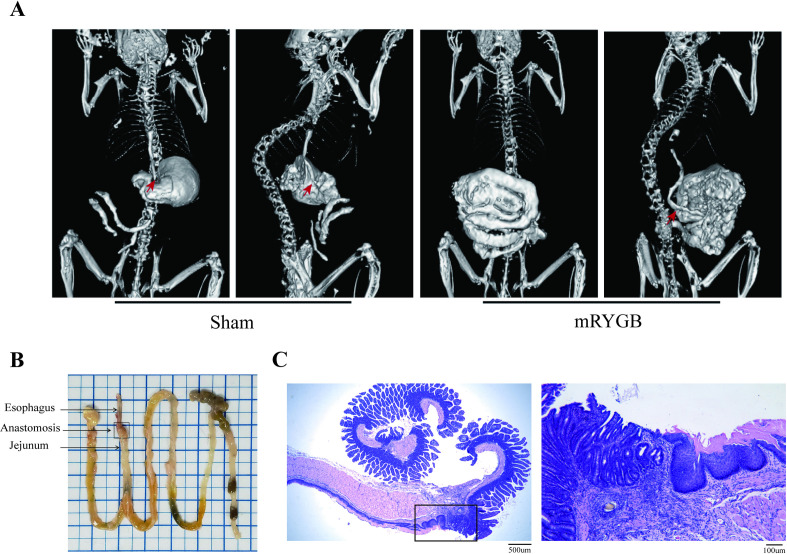
The status of gastroesophageal anastomosis after mRYGB surgery. A, PET-CT representative diagram of the esophagojejunal anastomotic site. The red arrow points towards the esophagojejunal anastomosis site. B, Representative morphological images of the esophagojejunal anastomotic site. C, Representative images of hematoxylin–eosin staining esophagojejunal anastomosis site of the mRYGB mice.

To investigate the healing of the anastomosis after using an auxiliary device, we used 5% barium powder as a gastrointestinal contrast agent to gavage the aforementioned mice 3–4 weeks after surgery and obtained reconstructed images of the gastrointestinal tract after Sham surgery and mRYGB surgery through real-time CT scanning. CT imaging examination showed that the stomach of Sham surgical mice was visible below the esophagus, and most of the barium meal remained in the stomach. Moreover, after mRYGB surgery, the barium meal of the mice quickly entered the small intestine, and there was no leakage or obvious narrow incisions at the anastomotic site (Fig 2A). In order to examine the anastomotic condition, we observed the gastrointestinal morphology and HE staining of mice 8 weeks after mRYGB surgery (Figs 2B and 2C). There was no infiltration around the anastomosis. The image showed a natural transition between the squamous epithelium of the esophagus and the columnar epithelium of the jejunum (Fig 2C). The above representative data have been repeatedly verified and have good repeatability, which fully demonstrates that our XL anastomotic device combined with mRYGB could effectively avoid postoperative complications and facilitate early postoperative recovery.

### The mRYGB surgery improves the quality of life in mice

**Fig 3:**
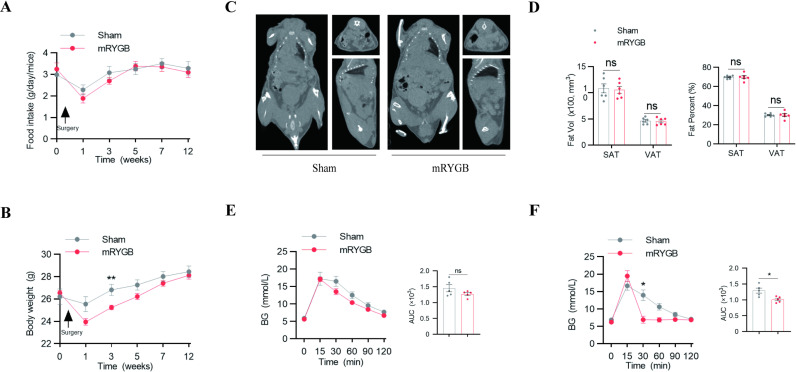
RYGB combined with anastomotic devices (mRYGB) improves the quality of life in mice. A and B, The food intake (A) and time courses of body weight (B) in sham and mRYGB surgery mice (n = 6). C, Representative Micro-CT image of sham and mRYGB surgery mice. D, Visceral and subcutaneous fat volume and relative volume to body weight in mice after sham and mRYGB surgery (n = 6). E and F, Blood glucose levels during intraperitoneal and oral glucose tolerance tests performed on sham and mRYGB surgery mice(n = 5). The areas under the curve of each group are shown on the right. The data are presented as the mean ± SEM. *p < 0.05, **p < 0.01.

The food intake of the mRYGB group at 4 weeks returned to the same level as that of the Sham group (Fig 3A). In addition, after mRYGB surgery, mice quickly regained weight after experiencing weight loss caused by surgical trauma (Fig 3B). Then, micro-CT was used to analyze the body fat of mice, which showed no significant difference in visceral and subcutaneous fat content between the mRYGB group and the Sham group (Figs 3C and 3D). This also confirmed that the mRYGB procedure almost avoids the occurrence of surgical complications. In terms of glucose tolerance, there was no difference in IPGTT between the two groups; however, in OGTT, the blood glucose levels of mRYGB-treated mice showed a rapid increase and then a rapid decrease curve (Figs 3E and 3F). It confirmed that mRYGB procedures improved glucose tolerance. In summary, the mRYGB procedure was effective, and the long-term survival of mice was not affected.

## Discussion

We combined our own years of practical experience to optimize a surgical method that can effectively avoid postoperative complications and improve the success rate of RYGB surgery in mice. By using an anastomotic auxiliary device composed of rigid and flexible parts, the problem of asymmetric esophagojejunal anastomosis can be effectively solved, and the quality of anastomosis can be improved. Based on our observations of mRYGB-treated mice, the mRYGB procedure almost completely avoids the problems of esophagojejunal anastomotic leakage and anastomotic stenosis and improves the long-term quality of life in mice.

Rodents are increasingly being used to study the mechanism of metabolic surgery. Mice hold significant importance in biomedical research due to the extensive availability of genetically engineered models. RYGB surgery assisted by the anastomotic device, as reported in this article, represents a straightforward, reproducible, and stable modeling approach. This method offers crucial technical support for the investigation of the mechanisms underlying metabolic surgery. At present, metabolic surgery represents the most effective therapeutic approach for both obesity and type 2 diabetes. As a result, there exists an immediate imperative to conduct investigations into its mechanism of action using experimental animal models [17]. However, as Stevenson reported, among the 82 cases presented in the published report, the mortality rate of RYGB surgery reached 29% [10]. This high mortality rate consequently renders the interpretation of the results challenging. At present, one model, developed by Hao et al. and independently replicated by Stevenson et al., which is based on anastomosing a small gastric pouch with the jejunum, is characterized by low mortality and a low incidence of complications. Importantly, low mortality does not represent the success of surgery. And postoperative trauma and complications result in poor repeatability and instability of subsequent research results, especially non-fatal mild complications such as mild anastomotic leakage or stenosis. Although this kind of complication will not cause early mouse death, it will affect the quality of life in mice and the experimental results, violating the principle of animal welfare [18].The anastomotic assistant device in RYGB operation can reduce the diameter deviation of the expanded esophagus and jejunum, which makes it more convenient for the operator to suture and avoids anastomotic stenosis. With this method, surgical strike, bleeding, anastomotic leaks, anastomotic obstruction, and the incidence of anemia were significantly decreased. Compared to our earlier modeling approaches which did not make use of this device, this method is clearly superior. Nevertheless, in comparison with the method proposed by Hao et al., there remains scope for further enhancement. Significantly, it offers a surgical methodology for researchers who are interested in metabolic surgery.

As the number of clinical metabolic surgeries increases, the development of unified quality and safety standards is accelerating. Correspondingly, the method for constructing RYGB should be associated with extremely low mortality and complication rates, accompanied by high reproducibility. The key is to try to avoid postoperative complications and ensure animal welfare. Therefore, here we describe a simple device that significantly effectively avoids the problems of anastomotic leakage and stenosis in mice and improve long-term quality of life.

## Conclusion

We have invented a gastrointestinal anastomosis auxiliary device consisting of a rigid front end and a flexible rear end. By using this device, the surgical method of RYGB has been modified to address issues such as difficulty in modeling and postoperative complications.

## Supporting information

S1 File Survival raw data. (XLSX)

S2 File Complication raw data. (XLSX)

S3 File Food intake raw data. (XLSX)

S4 File Body weight raw data. (XLSX)

S5 File Fat raw data. (XLSX)

S6 File IPGTT raw data. (XLSX)

S7 File OGTT raw data. (XLSX)
